# One-year outcome of a lumboperitoneal shunt in older adults with idiopathic normal pressure hydrocephalus

**DOI:** 10.3389/fsurg.2022.977123

**Published:** 2022-09-21

**Authors:** Xuhao Fang, Yao Deng, Xinxin Xu, Weiquan Shu, Feng Tang, Shihong Li, Ting Zhu, Li Zhang, Ping Zhong, Renling Mao

**Affiliations:** ^1^ Department of Neurosurgery, Huadong Hospital Affiliated to Fudan University, Shanghai, China; ^2^ Clinical Research Center for Geriatric Medicine, Huadong Hospital Affiliated to Fudan University, Shanghai, China; ^3^ Department of Neurosurgery, Huashan Hospital Affiliated to Fudan University, Shanghai, China; ^4^ Department of Radiology, Huadong Hospital Affiliated to Fudan University, Shanghai, China; ^5^ Department of Rehabilitation Medicine, Huadong Hospital Affiliated to Fudan University, Shanghai, China; ^6^ Department of Neurology, Huadong Hospital Affiliated to Fudan University, Shanghai, China

**Keywords:** idiopathic normal pressure hydrocephalus, lumboperitoneal shunt, functional improvement, symptomatic improvement, complication

## Abstract

**Background:**

Lumboperitoneal shunt (LPS) is now an effective surgical modality for idiopathic normal pressure hydrocephalus (iNPH), but there is still a lack of clinical data on LPS in older adult iNPH patients in China. We aim to report the shunt effect and the complications of older adult iNPH patients treated with LPS at a single center in Shanghai, China.

**Methods:**

We conducted a retrospective study among adults over 60 years old who were diagnosed as iNPH and treated with LPS from September 2016 to December 2020. The shunt effect was evaluated from two dimensions of functional and symptomatic improvement 3 months and 1 year after surgery, respectively. The potential factors related to the shunt effect one year after surgery were explored by comparing the effect between different subgroups and conducting multivariate logistic regression analysis.

**Result:**

A total of 85 patients were included in this study, ranging from 60 to 93 years old, with an average age of 74.7. The function and symptoms were better both 3 months and 1 year after surgery than before (*P *< 0.001). At the 1-year postoperation follow-up, the functional and symptomatic improvement rates were 72.9% and 90.6%, respectively. The symptomatic improvement rates of gait, urination, and cognition were 74.1%, 72.9%, and 60.0%, respectively. Multivariate logistic regression analysis showed that improvement in function was much more possible in patients with less than 24 months from symptom onset to surgery (OR* *= 24.57, *P *< 0.001) and those with disproportionately enlarged subarachnoid-space hydrocephalus (OR* *= 5.88, *P *= 0.048); improvement in gait was also more possible in patients with less than 24 months from symptom onset to surgery (OR* *= 5.29, *P *= 0.017); improvement in urination was more possible in patients with diabetes (OR* *= 4.76, *P *= 0.019), and improvement in cognition was more possible in patients with preoperative modified Rankin scale level lower than 4 (OR* *= 3.51, *P *= 0.040). Minor operation-related complications were seen in 27 patients (31.8%) and severe complications in 6 patients (7.1%).

**Conclusion:**

LPS could improve the function and symptoms of older adult iNPH patients. Early detection, diagnosis, and treatment of the disease could improve the shunt effect of the patients. Older adult iNPH patients with higher age ranges could achieve comparable shunt results compared with younger adults.

## Introduction

Normal pressure hydrocephalus (NPH) was first reported by Hakim and Adams in 1965 (1). The typical clinical manifestations of NPH are gait disorder, cognitive impairment, and urinary dysfunction. The brain images of patients showed ventricular enlargement, while the cerebrospinal fluid (CSF) pressure measured by lumbar puncture was normal (2). These typical symptoms can be partially or largely relieved by temporarily draining the CSF from the lumbo-subarachnoid space or by diverting the CSF to atrium or abdominal cavity (3).

NPH could be divided into secondary normal pressure hydrocephalus (sNPH) and idiopathic normal pressure hydrocephalus (iNPH) (4). The formation of sNPH has clear etiology (traumatic brain injury, subarachnoid hemorrhage, intracranial infection, etc.), and it can occur at all adult stages. Unlike sNPH, iNPH tends to occur in older adults, and no specific causes can be identified to fully explain the formation of NPH in the patient. Among the three NPH cases reported by Hakim and Adams, two were found to be secondary to brain head trauma and one idiopathic (1).

Generally, the prevalence of iNPH varies from 1.4% to 3.7% in a population-based setting over the age of 65 years (5–8) and reaches as high as 5.9%–8.9% over the age of 80 years (5, 6), showing a tendency of surging with increasing age. The prevalence also has a trend of increasing year by year from 0.04% in 2007 to 0.2% in 2017 in an inpatient setting over 60 years of age in the United States (9). There have been no relevant epidemiological data in China until now. It is speculated that there might be millions of older adults suffering from iNPH in the country if we refer to the existing international data.

For a long period, ventriculoperitoneal shunting (VPS) had been identified as the most common applied surgical procedure for iNPH patients in most countries of the world (3, 10). But this situation had seen some changes. A study conducted in the United States in 2020 showed that 2,115 iNPH patients who received a lumboperitoneal shunt (LPS) in the United States between 2007 and 2017 accounted for 15.5% of the 11,363 patients who underwent surgery for iNPH over the same period, making it the second-largest shunt after the VPS (11). In Japan, in 2017, 43.2% and 55.1% of iNPH patients received VPS and LPS treatment, respectively (12), and LPS had surpassed VPS to be number one in this country. Nowadays, more and more investigators suggest that LPS might be the alternative or first-line treatment option for this disease (13–16). However, the clinical data of LPS treatment for older adult iNPH patients from China are still scanty. We therefore aim to report the shunt effect and complications of LPS for older adult iNPH patients in the single center of Huadong Hospital, Shanghai, China.

## Methods

This is a descriptive study that retrospectively analyzed the information of older adult patients with iNPH before LPS and within one year after operation.

### Patients

**Figure 1:**
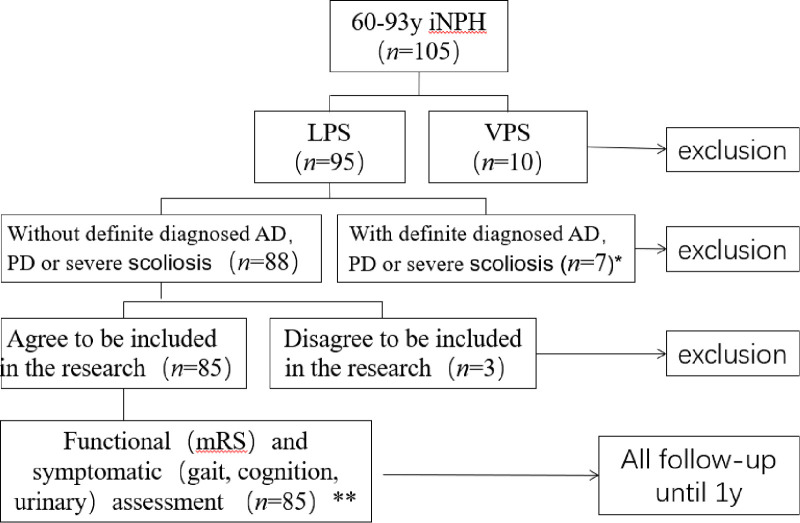
Flow chart of the study and the patient numbers from inclusion to final analysis.

Adults over 60 years old who were diagnosed as iNPH and treated with LPS from September 2016 to December 2020 in the Neurosurgical Department of Huadong Hospital affiliated to Fudan University in Shanghai, China, were included in this study. Diagnosis of the disease was made mainly according to the 2005 International iNPH Guidelines (2). The inclusion criteria were as follows: (1) age 60 years or older; (2) presence of gait/balance disorder and one or two other symptoms of the triad; (3) head computerized tomography (CT) and/or magnetic resonance imaging (MRI) showing ventriculomegaly indicated by an Evans’ index (EI) of >0.3; (4) absence of known disorders causing ventriculomegaly; (5) normal CSF opening pressure (≤20 cm H_2_O) and white blood cell (WBC) (WBC count ≤10 cells/ml); (6) positive response to the CSF Tap Test (17–20); (7) those who underwent LPS as the first CSF shunt surgery. The exclusion criteria were as follows: (1) Patients with secondary normal pressure hydrocephalus; (2) EI ≤ 0.3, although diagnosed as iNPH according to other index, such as z-Evans’ index (z-EI) (20); (3) with severe scoliosis; (4) diagnosed as Alzheimer's disease (AD) (21, 22) or Parkinson's Disease (PD) (23) combined with iNPH; (5) those who previously underwent VPS or LPS. A flow chart of the study and the patient numbers from inclusion to final analysis are shown in Figure 1.

The study was conducted in line with the Good Clinical Practice Guidelines and the World Medical Association Declaration of Helsinki (2002). All subjects were informed beforehand and they agreed to participate in the research plan approved by the ethics committee of Huadong Hospital Affiliated to Fudan University (2017K027).

### Surgery

The LPS shunting system included the Lumbo-Peritoneal Catheter Set (LPS50, SOPHYSA, Inc., ORSAY, France) and the MRI-resistant adjustable (five-position) valve with reservoir (SPVA, SOPHYSA, Inc., ORSAY, France). When performing LPS, L3–4/L2–3 puncture with fine needle guidance was performed and then the proximal catheter was inserted 5–10 cm into the subarachnoid space toward the head. The distal catheter was inserted into the abdominal space through the reverse McBurney's point of the left lower abdomen and connections were made between the catheters and the valve. The valve was placed under the skin beside the anterior superior iliac spine.

### Data collection and patient assessment

**Figure 2:**
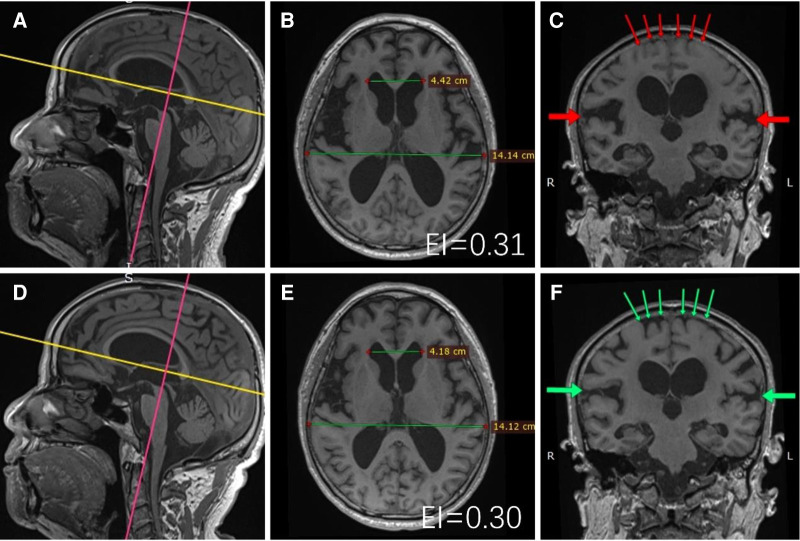
A 72-year-old male patient underwent T1-weighted thin-layer head MRI scans before and after surgery. Multiplanar reconstructions of the data are shown above. Preoperative: (**B,C**) were reconstructed along the yellow and pink lines of (**A**), respectively; Postoperative: (**E,F**) were reconstructed along the yellow and pink lines of (**D**), respectively. EI measurements (EI is the ratio of the maximal distance of frontal horns to the maximal inner diameter of the skull on the same plane) were performed at the same transversal planes, the postoperative (**B**) EI decreased compared with that of preoperative (**E**); Signs of DESH were observed in the same coronal planes, the degree of postoperative convex subarachnoid space tightness (green thin arrows of **F**) was reduced compared with that of preoperative (red thin arrows of **C**), and the degree of postoperative Sylvian fissure enlargement [green thick arrows of (**F**)] was reduced compared with that of preoperative [red thick arrows of (**C**)]. The patient's preoperative mRS was level 4, which improved to level 1 one year after LPS surgery.

Clinical data such as age, gender, disease history, and medical treatment history upon admission of the subjects were collected and brain imaging information such as EI and the presence or absence of disproportionately enlarged subarachnoid hydrocephalus (DESH) was recorded Figure 2.

We evaluated the functional status of the patients by assessing the modified Rankin Scale (mRS) (24, 25) and evaluated the symptomatic status by assessing the iNPH grading scale (iNPHGS) (19, 26); in addition, gait disorder was evaluated by using the 3-m Timed Up and Go test (3 m-TUG) (27) and cognition impairment by the Mini-Mental State Examination (MMSE) (28) before surgery. All measurements on functional and symptomatic status were conducted at 3 months and 1 year after shunt surgery.

The operation-related complications were recorded and classified into severe complications (shunt infection, subdural hematoma requiring surgery, shunt malfunction requiring reoperation, radiculopathy requiring surgery) and minor complications (orthostatic headache or dizziness, subdural hematoma or hygroma requiring no surgery, radiculopathy requiring no surgery). Non-operation-related severe events including death for other causes were also recorded.

The improvement rate of function 1-year post operation was the primary endpoint; the improvement rate of function 3-month post operation and the improvement rates of symptoms at 3-month or 1-year follow-up time were the secondary endpoints.

### Definitions

Functional improvement was defined as improving ≥1 levels on mRS (10, 13, 18).

Symptomatic improvements were defined as improving ≥1 grade on the iNPHGS (per domain of gait, cognition and urination or its total), a decrease of more than 10% on the 3 m-TUG, or an increase of ≥3 points on the MMSE (10, 13, 18).

The response to a Tap Test was deemed as positive if any of the above mentioned was found within one week after CSF removal (14).

### Statistical analysis

Statistical analysis of the study was performed using SPSS version 22.0 (SPSS, IBM, USA) and SAS 9.4 (SAS Institute Inc. Cary, NC, United States). All continuous variables were tested by using the Kolmogorov–Smirnov test for normality assessment and shown in the form of mean and standard deviation if distributed normally and described by median (range of quartiles) if not. All categorical variables were shown as values and rates. Comparisons for continuous variables were performed by using the *t*-test or Wilcoxon test according to the normality, and the Wilcoxon signed-rank test was used to compare the paired data. Chi-square test, or Fisher's exact test, was used for all categorical variables. To rule out potential confounders, multivariate logistic regression models were designed. All statistical tests were two-tailed, and *P *< 0.05 was considered statistically significant.

## Results

A total of 85 patients were included in this study ranging in age from 60 to 93 years, with an average age of 74.7 years, with 54 males (59%) and 31 females (41%). Median duration from symptom onset to operation was 24 months. A total of 71 patients (83.5%) presented with a complete Hakim triad; 60 patients (70.6%) had a history of falls due to balance/gait disorders. Radiographically, median EI was 0.34 and DESH was found in 64 patients (75.3%). There was no significant difference between male and female patients (*P *> 0.05) (Table 1).

**Table 1:** Basic characteristics of research subjects.

Variables	Total	Male (*N *= 54)	Female (*N *= 31)	*t*/*Z*/*χ*^2^	*P-*value
Age (year)	74.7 ± 7.1	75.6 ± 7.2	73.2 ± 6.9	1.48	0.142^a^
BMI (kg/m^2^)				0.09	0.928^b^
<18.5	1 (1.2)	1 (1.9)	0 (0.0)		
18.5–23.9	25 (29.4)	16 (29.6)	9 (29.0)		
24.0–27.9	53 (62.4)	33 (61.1)	20 (64.5)		
>27.9	6 (7.1)	4 (7.4)	2 (6.5)		
Hypertension	50 (58.8)	36 (66.7)	14 (45.2)	3.76	0.053
Diabetes	35 (41.2)	21 (38.9)	14 (45.2)	0.32	0.572
Hyperlipidemia	31 (36.5)	18 (33.3)	13 (41.9)	0.63	0.428
Once treated as AD	15 (17.7)	7 (13.0)	8 (25.8)	2.24	0.135
Once treated as PD	7 (8.2)	6 (11.1)	1 (3.2)	—	0.414^c^
Tumble				1.45	0.147^b^
No	25 (29.4)	17 (31.5)	8 (25.8)		
Occasionally	40 (47.1)	28 (51.9)	12 (38.7)		
Often	20 (23.5)	9 (16.7)	11 (35.5)		
EI	0.34 (0.31,0.35)	0.34 (0.31, 0.36)	0.34 (0.31, 0.35)	−0.93	0.354^b^
DESH	64 (75.3)	39 (72.2)	25 (80.7)	0.75	0.386
Months from symptom onset to operation	24 (14,48)	28 (14,48)	18 (12, 36)	−1.01	0.312^b^

^a^ Using the t-test.

^b^ Using the Wilcoxon test.

^c^ Using Fisher's exact test, and the others using the chi-square test.

Data are n (%), mean (SD, Standard deviation), or Median (Q1, Q3).

BMI, body mass index; AD, Alzheimer’s disease; PD, Parkinson's disease; EI, Evans’ index; DESH, disproportionately enlarged subarachnoid-space hydrocephalus.

### Shunt effect of LPS

Functional and symptomatic indicators of the patients were better 3 months and 1 year after surgery than before surgery, and the differences were both statistically significant (*P *< 0.001). There were no significant differences in functional and symptomatic indicators between 1 year and 3 months after surgery (*P *> 0.05) (Table 2).

**Table 2:** Comparison of functional and symptomatic indicators of patients at different times [median (Q1, Q3)].

	Pre operation	Postoperation 3 month	Postoperation 1 year	*T*	*P-*value_1_	*T*	*P-*value_2_	*T*	*P-*value_3_
mRS	4 (3, 4)	2 (2, 3)	3 (2, 3)	1,224.0	<0.001	992.0	<0.001	−22.5	0.087
iNPHGS Gait	3 (3, 3)	2 (2, 2)	2 (2, 2)	1,114.0	<0.001	1,019.5	<0.001	0.0	1.000
3 m-TUG^a^	23.7 (19.9, 30.5)	16.3 (14.4, 19.4)	17.2 (13.2, 19.4)	1,374.0	<0.001	1410.0	<0.001	−143.0	0.454
iNPHGS Cognition	3 (2, 3)	2 (2, 3)	2 (2, 3)	395.0	<0.001	456.0	<0.001	19.5	0.213
MMSE	16 (10, 21)	22 (15, 25)	21 (16, 25)	1,216.0	<0.001	1,364.5	<0.001	7.5	0.965
iNPHGS Urination	2 (1, 3)	1 (0, 2)	1 (0, 1)	990.0	<0.001	991.5	<0.001	5.5	0.795
iNPHGS Total	8 (6, 9)	5 (4, 6)	5 (4, 6)	1,544.0	<0.001	1,553.0	<0.001	32.5	0.279

The Wilcoxon Signed-rank test was used to compare the differences between groups; P-value_1_ (preoperation vs. postoperation 3 month), P-value_2_ (preoperation vs. postoperation 1-year); P-value_3_ (postoperation 3-month vs. postoperation 1 year).

^a^ n = 75.

mRS, modified Rankin scale; iNPHGS, idiopathic normal pressure hydrocephalus grading scale; MMSE, Mini-Mental State Examination.

### Functional and symptomatic improvement rates after surgery

At the 1-year postoperation follow-up, the functional (mRS) and symptomatic (iNPHGS total) improvement rates were 72.9% and 90.6%, respectively. In terms of the specific symptomatic improvement, the improvement rates of gait disturbances, urinary disorder, and cognitive impairment were 74.1%, 72.9%, and 60.0%, respectively.

The rate of functional (mRS) improvement one year after surgery decreased compared with three months after operation, while the improvement rate of symptomatic (iNPHGS total and cognition) increased overall, but none of the differences were statistically significant (*P *> 0.05) (Table 3).

**Table 3:** Comparison of improvement rates of symptoms and functions at different postoperative times [*n* (%)].

	mRS improved	iNPHGS gait improved	3 m-TUG improved^a^	iNPHGS cognition improved	MMSE improved	iNPHGS urination improved	iNPHGS total improved
Postoperation 3-month	69 (81.2)	66 (77.7)	65 (86.7)	39 (45.9)	46 (54.1)	62 (72.9)	76 (89.4)
Postoperation 1 year	62 (72.9)	63 (74.1)	67 (89.3)	42 (49.4)	51 (60.0)	62 (72.9)	77 (90.6)
*χ* ^2^	1.63	0.29	0.14	0.21	0.60	0.00	0.07
*P*-value	0.202	0.591	0.713	0.645	0.439	1.000	0.798

^a^ n = 75.

mRS, modified Rankin scale; iNPHGS, idiopathic normal pressure hydrocephalus grading scale; 3 m-TUG, 3-m Timed Up and Go test; MMSE, Mini-Mental State Examination.

### Analysis of related factors of improvement rate one year after surgery

The functional and symptomatic improvement rates in different subgroups at 1 year after operation were compared, and the results are shown in Table 4. From a functional point of view, the improvement rate in the mRS level was higher in patients with months ≤24 from symptom onset to operation than in patients >24 months (*P *< 0.05); from a symptomatic point of view, the improvement rates of iNPHGS total, 3m-TUG, MMSE, and urination disorder were not statistically different between any different groups (*P *> 0.05), but the improvement rate of gait disturbances was higher in patients with a history of hyperlipidemia or those with months ≤24 from symptom onset to operation. The improvement rate of cognitive impairment was also higher in patients with a history of hyperlipidemia (Table 4).

**Table 4:** Functional and symptomatic improvement rates in different subgroups 1 year after operation [*n* (%)].

	mRS improved	Gait disturbances improved	3 m-TUG improved^a^	Cognitive impairment improved	MMSE improved	Urination disorder improved	iNPHGS total score improved
Gender							
Male	22 (71.0)	22 (71.0)	22 (91.7)	16 (51.6)	16 (51.6)	23 (74.2)	26 (83.9)
Female	40 (74.1)	41 (75.9)	45 (88.2)	26 (48.2)	35 (64.8)	39 (72.2)	51 (94.4)
*χ*^2^	0.10	0.25	0.00	0.09	1.43	0.04	1.49
*P*-value	0.756	0.615	0.962^b^	0.758	0.232	0.844	0.222^b^
Age							
≤75 years	34 (70.8)	35 (72.9)	37 (86.1)	20 (41.7)	25 (52.1)	33 (68.8)	41 (85.4)
>75 years	28 (75.7)	28 (75.7)	30 (93.8)	22 (59.5)	26 (70.3)	29 (78.4)	36 (97.3)
*χ*^2^	0.25	0.08	0.48	2.65	2.88	0.98	2.21
*P*-value	0.618	0.773	0.490^b^	0.104	0.090	0.322	0.138^b^
Hypertension							
No	26 (74.3)	25 (71.4)	26 (83.9)	19 (54.3)	22 (62.9)	26 (74.3)	31 (88.6)
Yes	36 (72.0)	38 (76.0.)	41 (93.2)	23 (46.0)	29 (58.0)	36 (72.0)	46 (92.0)
*χ*^2^	0.05	0.22	0.82	0.57	0.20	0.05	0.02
*P*-value	0.815	0.636	0.365^b^	0.452	0.653	0.815	0.877^b^
Diabetes							
No	34 (68.0)	35 (70.0)	39 (86.7)	25 (50.0)	29 (58.0)	33 (66.0)	44 (88.0)
Yes	28 (80.0)	28 (80.0)	28 (93.3)	17 (48.6)	22 (62.9)	29 (82.9)	33 (94.3)
*χ*^2^	1.50	1.07	0.29	0.02	0.20	2.96	0.36
*P*-value	0.220	0.300	0.593^b^	0.897	0.653	0.085	0.549^b^
Hyperlipidemia							
No	36 (66.7)	35 (64.8)	41 (87.2)	21 (38.9)	31 (57.4)	42 (77.8)	48 (88.9)
Yes	26 (83.9)	28 (90.3)	26 (92.9)	21 (67.7)	20 (64.5)	20 (64.5)	29 (93.6)
*χ*^2^	2.95	6.68	0.14	6.56	0.41	1.75	0.10
*P*-value	0.086	0.010	0.707^b^	0.010	0.520	0.185	0.747^b^
BMI							
≤23.9	19 (73.1)	20 (76.9)	19 (86.4)	13 (50.0)	18 (69.2)	18 (69.2)	23 (88.5)
24.0-27.9	39 (73.6)	38 (71.7)	44 (91.7)	26 (49.1)	32 (60.4)	38 (71.7)	48 (90.6)
>27.9	4 (66.7)	5 (83.3)	4 (80.0)	3 (50.0)	1 (16.7)	6 (100.0)	6 (100.0)
*χ*^2^	0.13	0.53	—	0.01	5.62	2.45	0.76
*P*-value	0.937	0.766	0.462^c^	0.997	0.060	0.294	0.683
Once treated as AD							
No	52 (74.3)	53 (75.7)	57 (89.1)	35 (50.0)	41 (58.6)	53 (75.7)	65 (92.9)
Yes	10 (66.7)	10 (66.7)	10 (90.9)	7 (46.7)	10 (66.7)	9 (60.0)	12 (80.0)
*χ*^2^	0.08	0.16	0.00	0.05	0.34	0.85	1.12
*P*-value	0.778^b^	0.688^b^	1.000^b^	0.815	0.561	0.356^b^	0.289^b^
Once treated as PD							
No	58 (74.4)	59 (75.6)	62 (89.9)	41 (52.6)	46 (59)	57 (73.1)	70 (89.7)
Yes	4 (57.1)	4 (57.1)	5 (83.3)	1 (14.3)	5 (71.4)	5 (71.4)	7 (100.0)
*χ*^2^	0.29	0.38	—	2.39	0.06	0.00	—
*P*-value	0.591^b^	0.535^b^	0.504^c^	0.122^b^	0.809^b^	1.000^b^	1.000^c^
Tumble							
No	19 (76.0)	18 (72.0)	19 (82.6)	10 (40.0)	14 (56.0)	14 (56.0)	23 (92.0)
Occasionally	30 (75.0)	29 (72.5)	33 (89.2)	21 (52.5)	22 (55.0)	32 (80.0)	37 (92.5)
Often	13 (65.0)	16 (80.0)	15 (100.0)	11 (55)	15 (75.0)	16 (80.0)	17 (85.0)
*χ*^2^	0.84	0.47	2.88	1.29	2.46	5.15	0.96
*P*-value	0.656	0.789	0.237^b^	0.525	0.293	0.076	0.618
EI							
<0.34	32 (69.6)	34 (73.9)	36 (90.0)	23 (50.0)	31 (67.4)	34 (73.9)	42 (91.3)
≥0.34	30 (76.9)	29 (74.4)	31 (88.6)	19 (48.7)	20 (51.3)	28 (71.8)	35 (89.7)
*χ*^2^	0.58	0.00	0.00	0.01	2.28	0.05	0.00
*P*-value	0.447	0.963	1.000^b^	0.906	0.131	0.827	1.000^b^
DESH							
No	13 (61.9)	13 (61.9)	14 (77.8)	9 (42.9)	14 (66.7)	13 (61.9)	19 (90.5)
Yes	49 (76.6)	50 (78.1)	53 (93.0)	33 (51.6)	37 (57.8)	49 (76.6)	58 (90.6)
*χ*^2^	1.72	2.17	1.92	0.48	0.52	1.72	0.00
*P*-value	0.190	0.141	0.166^b^	0.489	0.472	0.190	1.000^b^
mRS preoperation							
<4	29 (82.9)	29 (82.9)	30 (88.2)	21 (60.0)	21 (60.0)	22 (62.9)	32 (91.4)
*≥*4	33 (66.0)	34 (68.0)	37 (90.2)	21 (42.0)	30 (60.0)	40 (80.0)	45 (90.0)
*χ*^2^	2.96	2.37	0.00	2.67	0.00	3.07	0.00
*P*-value	0.085	0.124	1.000^b^	0.102	1.000	0.080	1.000^b^
Months from symptom onset to operation							
≤24	40 (93.0)	37 (86.1)	41 (95.4)	22 (51.2)	25 (58.1)	29 (67.4)	41 (95.4)
>24	22 (52.4)	26 (61.9)	26 (81.3)	20 (47.6)	26 (61.9)	33 (78.6)	36 (85.7)
*χ*^2^	17.78	6.46	2.49	0.11	0.13	1.33	0.00
*P*-value	<0.001	0.011	0.115^b^	0.744	0.723	0.248	1.000^b^

^a^ n = 75.

^b^ Tested by using the continuity adjusted Chi-square test.

^c^ Tested by using Fisher's exact test.

The differences between groups were tested by using the Chi-square test.

mRS, modified Rankin scale; iNPHGS, idiopathic normal pressure hydrocephalus grading scale; 3 m-TUG, 3-m Timed Up and Go test; MMSE, Mini-Mental State Examination; BMI, body mass index; AD, Alzheimer’s disease; PD, Parkinson's disease; EI, Evans’ index; DESH, disproportionately enlarged subarachnoid-space hydrocephalus.

Multivariate logistic regression was performed to explore the factors affecting the effect of shunt, with the improvement in function or symptoms as the dependent variable, variables including diabetes, hyperlipidemia, and months from symptom onset to surgery as independent variables, and once treated as AD or not, DESH, and preoperative mRS as adjusted variables. The results are shown below.

First, the improvement of mRS was statistically correlated with the number of months from symptom onset to surgery. Patients with less than 24 months from symptom onset to operation were 11.11 times more likely to show improvement of mRS than those with more than 24 months (*P *< 0.001). Second, the improvement of gait disorder was also statistically correlated with the months from symptom onset to surgery (OR* *= 3.53, *P *= 0.025); in addition, patients with a history of hyperlipidemia was more likely to show improvement of mRS (OR* *= 4.64, *P *= 0.026).Third, improvement in urinary dysfunction was statistically associated with the presence or absence of diabetes. Improvement in urinary dysfunction after surgery was 3.13 times greater in diabetic patients than in nondiabetic patients (*P *= 0.047). The last one, improvement of cognitive impairment, was statistically correlated with a history of hyperlipidemia (OR* *= 3.45, *P *= 0.011). In addition, there was no statistical correlation between the improvement of MMSE, 3 m-TUG, and iNPHGS total and other factors after a similar analysis in this study.

The variables that may affect the functional and symptom improvement were incorporated into different models for analysis, and the results were not significantly different (Table 5).

**Table 5:** Multivariate logistic regression analysis of functional and symptom improvements at 1 year after operation.

	mRS improved		Gait disturbances improved		Urination disorder improved		Cognitive impairment improved	
	OR (95% CI)	*P*-value	OR (95% CI)	*P*-value	OR (95% CI)	*P*-value	OR (95% CI)	*P*-value
Model 1								
Diabetes	1.50 (0.47, 4.77)	0.496	1.30 (0.43, 3.95)	0.644	3.22 (1.05, 9.89)	0.041*	0.76 (0.30, 1.92)	0.564
Hyperlipidemia	2.28 (0.66, 7.85)	0.190	4.64 (1.20, 17.95)	0.026*	0.44 (0.16, 1.25)	0.125	3.45 (1.33, 8.97)	0.011*
≤24 months from symptom onset to operation	11.11 (3.03, 50.00)	<0.001*	3.53 (1.17, 10.65)	0.025*	0.53 (0.19, 1.46)	0.218	1.04 (0.43, 2.56)	0.927
Model 2								
Diabetes	1.50 (0.46, 4.82)	0.499	1.35 (0.44, 4.19)	0.601	3.13 (1.02, 9.65)	0.047*	0.75 (0.3, 1.91)	0.547
Hyperlipidemia	2.4 (0.69, 8.38)	0.171	5.04 (1.27, 20.01)	0.022*	0.46 (0.16, 1.31)	0.147	3.53 (1.35, 9.22)	0.010*
≤24 months from symptom onset to operation	11.11 (2.94, 50.00)	<0.001*	3.45 (1.11, 10.00)	0.032*	0.48 (0.17, 1.39)	0.174	1.01 (0.41, 2.50)	0.984
DESH	1.97 (0.58, 6.74)	0.279	2.36 (0.72, 7.66)	0.154	2.12 (0.68, 6.58)	0.192	1.53 (0.53, 4.37)	0.430
Model 3								
Diabetes	1.58 (0.49, 5.16)	0.446	1.30 (0.42, 3.98)	0.649	3.24 (1.04, 10.04)	0.042*	0.76 (0.3, 1.94)	0.566
Hyperlipidemia	2.05 (0.58, 7.26)	0.266	4.33 (1.1, 16.99)	0.036*	0.50 (0.17, 1.45)	0.203	3.25 (1.23, 8.57)	0.017*
≤24 months from symptom onset to operation	10.81 (2.82, 41.42)	0.001*	3.28 (1.07, 10.05)	0.037*	0.57 (0.20, 1.62)	0.290	0.93 (0.37, 2.34)	0.877
mRS level <4 preoperation	1.68 (0.50, 5.70)	0.403	1.53 (0.48, 4.87)	0.467	0.48 (0.17, 1.36)	0.167	1.86 (0.73, 4.73)	0.194
Model 4								
Diabetes	1.48 (0.46, 4.76)	0.514	1.31 (0.43, 3.99)	0.637	3.69 (1.15, 11.84)	0.028*	0.76 (0.30, 1.92)	0.562
Hyperlipidemia	2.31 (0.66, 8.06)	0.188	4.60 (1.18, 17.90)	0.028*	0.38 (0.13, 1.13)	0.083	3.46 (1.33, 9.03)	0.011*
≤24 months from symptom onset to operation	11.70 (3.04, 45.01)	<0.001*	3.49 (1.14, 10.67)	0.028*	0.45 (0.15, 1.30)	0.138	1.05 (0.42, 2.60)	0.920
Once treated as AD	1.11 (0.28, 4.42)	0.883	0.92 (0.24, 3.45)	0.896	0.3 (0.08, 1.12)	0.072	1.04 (0.32 3.38)	0.952

* P < 0.05.

Univariate analysis found that improvement rates were different between patients with or without hyperlipidemia and patients with different months from symptom onset to operation. On this basis, we have established four models: Model 1 was adjusted by hyperlipidemia, diabetes, months from symptom onset to operation; Model 2, Model 3, and Model 4 added the variables of DESH, mRS level preoperation, and once treated as AD, respectively.

OR, odds ratio; CI, confidence interval; DESH, disproportionately enlarged subarachnoid-space hydrocephalus; mRS, modified Rankin score; AD, Alzheimer’s disease.

### Operation-related complications and other severe adverse events

No operation-related death occurred in this case series. Operation-related severe complications occurred in six patients (7.1%), including two with shunt infection, two with subdural hematoma requiring surgical treatment and two with shunt malfunction. Operation-related minor complications occurred in twenty-seven patients (31.8%). Orthostatic dizziness, orthostatic headache and subdural effusions are the most common seen minor complications that mainly occurred within one month after operation (Table 6).

**Table 6:** Operation-related complications [*n* (%)].

	From operation to 1 month post operation	2–3 months post operation	4–12 months post operation	Total
Severe	2 (2.4)	1 (1.2)	3 (3.5)	6 (7.1)
Shunt infection	2 (2.4)	0 (0.0)	0 (0.0)	2 (2.4)
Subdural hematoma requiring surgery	0 (0.0)	1 (1.2)	1 (1.2)	2 (2.4)
Shunt malfunction	0 (0.0)	0 (0.0)	2 (2.4)	2 (2.4)
Radiculopathy requiring surgery	0 (0.0)	0 (0.0)	0 (0.0)	0 (0.0)
Minor	20 (23.5)^a^	8 (9.4)^a^	7 (8.2)^a^	27 (31.8)^a^
Orthostatic headache	6 (7.1)	2 (2.4)	3 (3.5)	10 (11.8)^a^
Orthostatic dizziness	16 (18.8)	5 (5.9)	6 (7.1)	23 (27.1)^a^
Subdural effusion	2 (2.4)	2 (2.4)	2 (2.4)	6 (7.1)
Subdural hematoma requiring no surgery	2 (2.4)	1 (1.2)	0 (0.0)	3 (3.5)
Radiculopathy requiring no surgery	3 (3.5)	1 (1.2)	0 (0.0)	3 (3.5)^a^

^a^ Refers to the results obtained after removing the repeated calculation of different types and degrees of complications in the same patient.

Other serious adverse events including spontaneous intracerebral hemorrhage, traumatic brain injury, severe pneumonia and severe heart attack occurred in at least five patients (5.9%). Two of these patients died within one year after LPS surgery.

## Discussion

In recently published studies, more and more investigators comprehensively applied the mRS and iNPHGS as the main methods to assess the patients (10, 15, 18). Besides, 3m-TUG and MMSE were used as an objective test for iNPH patients (10, 15, 18, 29). The same comprehensive assessment methodology was used in this study. In order to better evaluate the shunt effect, in our study, we propose dividing the shunt effect of every patient into functional improvement (expressed by mRS) and symptomatic improvement (expressed by iNPHGS).

The diagnostic criteria for iNPH in this study mainly referred to the 2005 International iNPH Guidelines (2). At the same time, the 2012 Japanese iNPH Guidelines (19) were also taken into account. As a result, the diagnostic criteria for iNPH in this study should be stricter, and theoretically, the shunt effect of patients in this study would be higher than that of patients diagnosed and treated solely on the 2005 International Guidelines or the Japanese Guidelines.

Just as many previous case cohort studies (18, 30, 31) and epidemiological studies (6, 9, 12, 29, 32) had shown, our older adult iNPH cases were also showing a slight male predominance though without any significant difference. In addition, the baseline data for preoperative evaluation of concomitant diseases, symptom manifestations, imaging characteristics, and other related indicators of this study were similar to those of earlier studies (18, 29–31). Therefore, the results of this study are comparable to those of earlier studies with the same assessment methods and follow-up time.

Hashimoto et al. (18) conducted a multicenter prospective study that iNPH patients received VPS with the Codman Hakim Programmable Valve. The 1-year favorable outcome, here interpreted as the functional improvement rate, was 69%. Later in 2015, Kazui et al. (13) conducted a multicenter prospective randomized controlled study that iNPH patients received LPS with the Codman Hakim Programmable Valve with a Siphon-Guard. The overall 1-year favorable outcome, here interpreted as the functional improvement rate, was 63%. Miyajima et al. (33) made a comparison using historical control between the above-mentioned two studies and concluded that LPS shunt efficacy was comparable to that of VPS. Nakajima et al. (14) retrospectively reviewed an iNPH patient cohort that received LPS with the Strata NCS Valves and found a 1-year response rate, here interpreted as the functional improvement rate, of 64%. The functional improvement rate one year after LPS in our study is 72.9%, slightly higher than that of the above-mentioned studies. This result shows a better shunt effect of LPS in our center. A direct comparison of the functional improvement rate with that of several other previous studies is unreliable due to the different outcome assessment methods and/or different follow-up times (15, 16).

To identify factors that might influence the outcome of functional improvement, multivariate logistic regression analysis was conducted, and the results showed that the functional improvement rate in patients less than 24 months from symptom onset to operation was much higher than that in the others. The results suggest that efforts should be made to correctly diagnose and treat the disease as early as possible, so that shunt surgery can achieve the best therapeutic effect. As to imaging characteristics, the improvement rate was higher in patients with DESH than those without, but the improvement rates showed no significant difference between patients with larger ventricles (EI ≥ 0.34) or not (EI < 0.34). These imaging findings are concordant with those of many earlier studies (13, 18, 30, 31). An uncommon finding in our study was that the functional improvement rate in female patients was lower than that in male patients, which might indicate a disputable slight male predisposition of iNPH different from the female predisposition of AD (21, 22, 34).

Although the function of some patients did not improve, their symptoms did, which also met our expectations. The symptomatic improvements seen in our study were consistent with those in many earlier studies (13, 14, 18, 35). Gait dysfunction was the most obviously improved among the three domains. With regard to the other two domains, most studies (10) reported that the cognitive improvement rate was higher than that of urination, while some studies reported converse results (36). Our study result showed that the improvement rate of urinary dysfunction was higher than that of cognitive impairment.

The oldest patient who underwent LPS (Sep 2018) in this study was 93 years of age. His rehabilitation process was found to be good until a few months back (Feb 2022). This study showed that the postoperative functional improvement rate and symptomatic improvement rates in older iNPH patients with higher age ranges (>75 years) were not worse than those in older iNPH patients with lower age ranges (≤75 years). Clinicians may counsel patients and their families that an increasing age does not necessarily decrease the chances of a shunt being successfully implanted (37). This study result might suggest that older probable iNPH patients are more likely to be certain candidates for iNPH after surgery, while younger ones are more likely to be accompanied by other diseases. Anyway, older iNPH patients should not be unjustifiably excluded as shunting candidates (36, 38).

Apart from focusing on patient improvement, we need to pay close attention to surgical complications that patients may experience. As we know, complications can severely affect surgical outcomes, especially at an early postoperation stage (20). Severe complications were seen in six patients in this study. Of them, only one patient functionally improved, another one unchanged, the other four functionally deteriorated. Early detection and management of serious complications can reduce injuries, while delayed detection and management will increase injuries. Minor complications, such as orthostatic headache and dizziness are more common, but they can usually be relieved by conservative treatment (20, 39).

Although functional and/or symptomatic improvements are the most desired results, it should be noted that some patients experience no improvement either in terms of function or in terms of symptoms despite successful surgery. On closer examination, there are three possible scenarios. First, the rapid worsening of symptoms before surgery could have been prevented at a certain stage after surgery, which might be neglected by observers. Second, comorbidities, such as concealed cerebrovascular diseases in some patients, might lead to various exacerbations of symptoms and/or functional declines, thus canceling the positive effect that surgery brings with it (40). Third, the accelerated natural aging of very old patients could also counteract the supposed benefits of surgery, especially during the long-term follow-up (41). Because of the existence of these conditions, we need to conduct an in-depth and effective communication exercise with family members and patients before surgery.

In order to achieve better treatment results, we recommend: (1) Pay attention to the diagnosis, typical clinical symptoms, and imaging characteristics as these are the most important, otherwise wait and watch, rather than rushing to surgery; (2) Always emphasize the whole process management of aseptic technology; (3) Detect and treat serious complications as early as possible; (4) Provide timely treatment of minor complications, as otherwise, they easily lead to serious complications; (5) Strengthen follow-up and maintain good communication with patients and their family members.

This study has several limitations. Although this study prospectively collected case data from 2016 until the end of 2021, it is still classified as a retrospective case analysis study because there was no clinical trial registration. The study included a relatively limited case series from a single center. The observer did not use method of blindness , which may inevitably leads to bias. The study did not contain a control group, and thus the results could not be compared with those of other operational methods directly. It is also unfortunate that the above three undesired scenarios cannot be reflected in the existing functional and symptomatic evaluation system, which needs to be further optimized in the future. Nevertheless, we hope that the findings from this study will provide encouragement for further research into LPS as the primary treatment for iNPH in older adults in China.

## Conclusion

LPS could improve the function and symptoms of older adult iNPH patients. Early detection, diagnosis, and treatment of the disease could improve the shunt effect of the patients. Older adult iNPH patients with higher age ranges could achieve comparable shunt results compared with those with lower age ranges.

## Data Availability

The original contributions presented in the study are included in the article/Supplementary Material, and further inquiries can be directed to the corresponding authors.
